# Association between METTL14 gene polymorphisms and risk of ovarian endometriosis

**DOI:** 10.3389/fgene.2024.1460216

**Published:** 2025-01-03

**Authors:** Zijun Zhou, Youkun Jie, Xianyue Hu, Guange Chen, Yanjing Bao, Zhenbo OuYang, Liangzhi Wu, Tianyang Gao, Qiushi Zhang, Wenfeng Hua

**Affiliations:** ^1^ Department of Reproductive Medicine Center, The Affiliated Guangdong Second Provincial General Hospital of Jinan University, Guangzhou, Guangdong, China; ^2^ Department of Gynecology and Obstetrics, The Third People’s Hospital of Chengdu, The Affiliated Hospital of Southwest Jiaotong University, Chengdu, Sichuan, China; ^3^ Department of Pathology, Jiangxi Maternal and Child Health Hospital, Nanchang, Jiangxi, China; ^4^ The Second School of Clinical Medicine, Southern Medical University, Guangzhou, Guangdong, China; ^5^ Department of Gynecology, The Affiliated Guangdong Second Provincial General Hospital of Jinan University, Guangzhou, Guangdong, China; ^6^ Department of Gynecology and Obstetrics, The Second Affiliated Hospital and Yuying Children’s Hospital of Wenzhou Medical University, Wenzhou, Zhejiang, China; ^7^ Research Institute for Maternal and Child Health, The Affiliated Guangdong Second Provincial General Hospital of Jinan University, Guangzhou, Guangdong, China

**Keywords:** endometriosis, infertility, gene polymorphism, METTL14, Chinese population

## Abstract

**Background:**

Endometriosis, a prevalent chronic gynecological condition, is frequently associated with infertility and pelvic pain. Despite numerous studies indicating a correlation between epigenetic regulation and endometriosis, its precise genetic etiology remains elusive. Methyltransferase-like 14 (METTL14), a crucial component of the N6-methyladenosine (m^6^A) RNA methyltransferase complex and an RNA binding scaffold, is known to play a pivotal role in various human diseases. The possibility that single nucleotide polymorphisms (SNPs) in the METTL14 gene contribute to susceptibility of endometriosis has not been thoroughly investigated.

**Methods:**

We assessed the genotype frequencies of five potential functional METTL14 SNPs (rs298982 G>A, rs62328061A>G, rs9884978G>A, rs4834698C>T, and rs1064034A>T) in a Chinese population consisting of 458 patients with ovarian endometriosis and 462 healthy controls. We employed unconditional logistic regression and stratified analyses to evaluate their genotypic associations with the risk of ovarian endometriosis.

**Results:**

Among the five SNPs examined, we found that the rs298982 A allele was significantly associated with increased risk, whereas the rs62328061 G allele was linked to a decreased risk of ovarian endometriosis. Individuals harboring two unfavorable genotypes demonstrated a significantly elevated risk of ovarian endometriosis (adjusted odds ratio (AOR) = 1.57, 95% confidence interval (CI) = 1.16–2.13, *P* = 0.004) compared with those with no risk genotypes. Stratified analysis revealed the risk effect of rs298982 GA/AA genotypes in the gravidity≤1, parity≤1, rASRM stage I, and rASRM stage II + III + IVsubgroups. Haplotype analysis showed that individuals with the GATAA haplotype were at higher risk of ovarian endometriosis (AOR = 5.54, 95% CI = 1.63–18.87, *P* = 0.006), whereas the AGTTG haplotype exhibited protective effects (AOR = 0.55, 95% CI = 0.31–0.97, *P* = 0.039) compared with wild-type GACAG haplotype carriers. Additionally, Bayesian false discovery probability and false positive report probability analysis confirmed the robustness of the significant findings. Expression quantitative trait loci analysis revealed a significant association between the rs9884978 GA/AA genotypes and elevated METTL14 mRNA levels in fibroblasts and adrenal gland. Conversely, the rs298982 GA/GG genotypes were significantly associated with reduced METTL14 mRNA levels in the nucleus accumbens and frontal cortex.

**Conclusion:**

Our results demonstrate that METTL14 polymorphisms are associated with susceptibility to ovarian endometriosis among Chinese women.

## 1 Introduction

Endometriosis is a common and often undiagnosed disease affecting 5%–10% of women of reproductive age globally (Zondervan et al., 2018; Taylor et al., 2021). This disease is characterized by an estrogen-dependent chronic inflammatory process that primarily affects pelvic tissues, including the ovaries. The ectopic endometrial tissue undergoes menstrual cycle changes similar to the typically located endometrium but with no means of discharging the menstrual fluid. Clinical manifestations are mainly painful menstrual cramps, bleeding or spotting between menstrual periods, and infertility (Giudice and Kao, 2004; Zondervan et al., 2018; Bulun et al., 2019; Taylor et al., 2021).

There are many theories regarding the pathogenesis of endometriosis, among which the Sampson hypothesis of implanted endometrial cells following retrograde menstruation is widely accepted (Sampson, 1927; Lamceva et al., 2023). However, this theory can neither explain why as many as 90% of women experience menstrual retrograde but less than 10% develop endometriosis nor why endometriosis lesions develop outside the abdominal cavity (Halme et al., 1984; Lamceva et al., 2023). The biology of endometriosis is multifactorial, involving multiple inflammatory, immune, hormonal, environmental, and genetic factors (Vercellini et al., 2014; Rahmioglu et al., 2023; Mokhtari et al., 2024). As early as the 1940s, a genetic-related familial aggregation tendency was associated with the incidence of endometriosis that was later supported by research in the 1980s (Bischoff and Simpson, 2000; Zondervan et al., 2018). Studies on identical twins have found that approximately 50% of the risk of endometriosis is associated with genetic factors (Treloar et al., 1999; Saha et al., 2015).

Recent studies have shown that epigenetic modifications are key drivers of endometriosis development and progression (Wang et al., 2020b; Marquardt et al., 2023). Of these, N^6^-methyladenosine (m^6^A) modification of mRNAs has been reported to regulate the progression of endometriosis (Huang and Chen, 2023). In one study, Lin *et al.* found that increased expression of m^6^A demethylase ALKBH5 upregulates the expression of EZH2 and H3K27Me3 in the hypoxic microenvironment of endometriosis (Lin et al., 2024). Wang *et al.* reported that the m^6^A methylase METTL3 expression levels and global m^6^A RNA modifications are reduced in endometriosis, promoting viability, proliferation, migration, and invasion in endometrial stromal cells (Wang et al., 2023). Other studies found that the m^6^A methylase methyltransferase-like14 (METTL14) is downregulated in the ectopic endometrium compared with that in the eutopic endometrium, and METTL14 knockdown increases cell proliferation and invasion in primary eutopic stromal cells (Shen et al., 2023).

In recent years, there has been increasing focus on the genetic aspects of endometriosis. Notably, Sapkota *et al.* discovered five novel loci that harbor candidate genes involved in sex steroid pathways that are significantly associated with the risk of endometriosis (*P* < 5 × 10^−8^) through a meta-analysis of genome-wide association studies in 11 individual case-control datasets of European and Japanese ancestry (Sapkota et al., 2017). Furthermore, Méar *et al.* identified that the polymorphisms of IFNG (CA) repeat, GSTM1 null genotype, GSTP1 rs1695, WNT4 rs16826658, and WNT4 rs2235529 were significantly associated with endometriosis in a substantial cohort of patients with well-defined inclusion criteria by a systematic review and meta-analysis (Mear et al., 2020). Considering the critical role of the m^6^A modulator proteins in maintaining proper cellular functions, it is important to examine how genetic variations in the m^6^A methyltransferase complex may affect endometriosis risk. Our previous study showed that WTAP rs1853259 and rs7766006 were significantly associated with the risk of ovarian endometriosis (Wan et al., 2023b). However, to our knowledge, no published studies have investigated the role of single nucleotide polymorphisms (SNPs) of METTL14 gene in the susceptibility to endometriosis. To fill this research gap, we genotyped five selected potential functional SNPs of the METTL14 gene and investigated their associations with the risk of endometriosis in a Chinese population.

## 2 Methods

### 2.1 Study subjects

This case-control study included 458 patients and 462 controls recruited from Jiangxi Provincial Maternal and Child Health Hospital between January 2015 and June 2022. All the patients had been diagnosed with histologically proven ovarian endometriosis and had been staged according to the revised American Society for Reproductive Medicine (rASRM) classification system. The controls were healthy female volunteers who had been visiting the same hospital for health examinations who had no family history of endometriosis or malignant neoplasms. This study was approved by the Institutional Ethics Committee of the Jiangxi Provincial Maternal and Child Health Hospital, and written informed consent was obtained from all participants.

### 2.2 SNP selection and genotyping

The five potential functional METTL14 SNPs (rs9884978G>A, rs62328061A>G, rs4834698C>T, rs1064034A>T, and rs298982 G>A) were selected from previous studies (Zhuo et al., 2020). These SNPs had minor allele frequencies (MAFs) ≥ 0.05 and a low linkage disequilibrium (*R* ^2^ < 0.8) in the Chinese population. The QIAamp DNA FFPE Tissue Kit (Qiagen, Valencia, CA) and Genome TIANGEN Blood DNA Extraction Kit (TianGen Biotech, Beijing) were used to extract genomic DNA from paraffin-embedded tissue samples and peripheral blood samples according to the manufacturer’s protocol. Quantity and quality control analysis of genomic DNA was performed using a NanoDrop spectrophotometer (ThermoFisher Scientific, Waltham, MA) and gel electrophoresis. The genotyping of the rs298982 G>A was conducted using the TaqMan method. The remaining four SNPs were genotyped using the Agena Bioscience MassArray iPLEX platform (Agena Bioscience, SanDiego, CA), which was based on matrix-assisted laser desorption/ionization-time-of-flight (MALDI-TOF) mass spectrometry. The assay was performed by CapitalBio Technology (Beijing). The genotyping completion rate for all SNPs was more than 95%.

### 2.3 Statistical analysis

A goodness-of-fit χ2 test was performed to assess the Hardy-Weinberg equilibrium (HWE) of METTL14 gene SNPs in the controls. The differences between the demographic and clinical characteristics of the patients and controls were analyzed using the Student’s t-test for continuous variables and the χ2 test for categorical variables. Crude and adjusted odds ratios (AORs) and their 95% confidence intervals (CIs) were calculated by univariate and multivariate logistic regression models. Associations between the genotypes and the risk of ovarian endometriosis among subgroups by age, gravidity, parity, and rASRM stage were further evaluated by stratification analysis. Expression quantitative trait locus (eQTL) analysis was performed through the Genotype-Tissue Expression (GTEx) portal website (http://www.gtexportal.org/home/) to evaluate the potential associations between the SNPs and expression levels of the METTL14 gene (Carithers and Moore, 2015). The false positive report probability (FPRP) and Bayesian false discovery probability (BFDP) were used to evaluate the robustness of significant findings, which were described in detail in our previous study (Wan et al., 2023b). All statistical analyses were conducted using SAS 9.4 (SAS Institute Inc., Cary, NC). *P*< 0.05 was considered statistically significant.

## 3 Results

### 3.1 Participant demographic and clinical characteristics

Table 1 presents the clinical characteristics of 458 patients with ovarian endometriosis and 462 control participants. The mean ages of the patients and controls were 31.99 ± 6.27 and 30.84 ± 7.39, respectively. There were no statistically significant differences in age or parity between the patients and controls. However, the patients had fewer pregnancies than the controls (*P*= 0.043). According to rASRM classification, 339 (74.0%) patients had stage I endometriosis (minimal), 99 (21.6%) had stage II endometriosis (mild), 15 (3.3%) had stage III endometriosis (moderate), and 5 (1.1%) had stage IV endometriosis (severe).

**TABLE 1:** Frequency distribution of selected variables for ovarian endometriosis patients and controls.

Characteristic	Case (N = 458)	Control (N = 462)	*P*
Age (year)			
Mean ± SD	31.99 ± 6.27	30.84 ± 7.39	0.051^a^
Gravidity			
≤1	410 (89.5%)	393 (85.1%)	0.043^b^
>1	48 (10.5%)	69 (14.9%)	
Parity			0.093^b^
≤1	418 (91.3%)	406 (87.9%)	
>1	40 (8.7%)	56 (12.1%)	
rASRM stage^c^			
I	339 (74.0%)		
II	99 (21.6%)		
III	15 (3.3%)		
IV	5 (1.1%)		

^a^ Student’s t-test for distribution between patients and controls.

^b^ χ2 test for distribution between patients and controls.

^c^ rASRM, revised American Society for Reproductive Medicine staging system.

### 3.2 Associations between METTL14 polymorphisms and risk of ovarian endometriosis

The results of analysis of the association between METTL14 gene SNPs and ovarian endometriosis risk are shown in Table 2. All the SNPs followed HWE in the controls (*P*> 0.05), except for rs4834698 (*P* = 0.016). In the single-locus analysis, the rs62328061variant alleles were significantly associated with decreased ovarian endometriosis risk (AG vs. AA: AOR = 0.69, 95% CI = 0.51–0.93, *P* = 0.014; dominant model: AOR = 0.75, 95% CI = 0.57–0.99, *P* = 0.047). The rs9884978 variant alleles were strongly associated with reduced risk of ovarian endometriosis (AA vs. GG: crude OR = 0.52, 95% CI = 0.27–0.99, *P* = 0.047; recessive model: crude OR = 0.51, 95% CI = 0.27–0.96, *P* = 0.036). In contrast, an increased risk of ovarian endometriosis was observed with the rs298982 variant alleles (GA vs. GG: AOR = 1.46, 95% CI = 1.06–2.02, *P* = 0.022; AA vs. GG: AOR = 6.11, 95% CI = 1.11–33.54, *P* = 0.037; dominant model: AOR = 1.54, 95% CI = 1.12–2.12, *P* = 0.008; recessive model: AOR = 5.66, 95% CI = 1.03–31.15, *P* = 0.046; additive model: AOR = 1.57, 95% CI = 1.17–2.13, *P* = 0.003). However, no significant associations were observed between the remaining variant alleles and ovarian endometriosis risk.

**TABLE 2:** Association between METTL14 gene polymorphisms and ovarian endometriosis susceptibility.

Genotype	Cases (N = 458)	Controls (N = 462)	Crude OR (95% CI)	*P* ^a^	Adjusted OR (95% CI)	*P* ^b^
METTL14 rs9884978 G>A, HWE = 0.069						
GG	286 (62.4%)	288 (62.3%)	1.00		1.00	
GA	157 (34.3%)	145 (31.4%)	1.09 (0.83–1.44)	0.543	1.10 (0.83–1.46)	0.497
AA	15 (3.3%)	29 (6.3%)	**0.52 (0.27**–**0.99)**	**0.047**	0.58 (0.30–1.11)	0.100
Dominant			0.99 (0.76–1.30)	0.973	1.02 (0.78–1.34)	0.886
Recessive			**0.51 (0.27**–**0.96)**	**0.036**	0.56 (0.29–1.07)	0.078
Additive			0.91 (0.73–1.14)	0.419^c^	0.94 (0.75–1.18)	0.600^c^
METTL14 rs62328061 A>G, HWE = 0.436						
AA	328 (71.6%)	301 (65.2%)	1.00		1.00	
AG	109 (23.8%)	147 (31.8%)	**0.68 (0.51**–**0.91)**	**0.010**	**0.69 (0.51**–**0.93)**	**0.014**
GG	21 (4.6%)	14 (3.0%)	1.38 (0.69–2.76)	0.367	1.40 (0.69–2.82)	0.348
Dominant			**0.74 (0.56**–**0.98)**	**0.035**	**0.75 (0.57**–**0.99)**	**0.047**
Recessive			1.54 (0.77–3.06)	0.221	1.56 (0.78–3.12)	0.214
Additive			0.85 (0.67–1.08)	0.178^c^	0.86 (0.68–1.09)	0.217^c^
METTL14 rs4834698 C>T, HWE = 0.016						
CC	116 (25.3%)	126 (27.3%)	1.00		1.00	
CT	230 (50.2%)	205 (44.4%)	1.22 (0.89–1.67)	0.218	1.21 (0.88–1.67)	0.233
TT	112 (24.5%)	131 (28.3%)	0.93 (0.65–1.33)	0.684	0.99 (0.69–1.42)	0.948
Dominant			1.11 (0.82–1.48)	0.503	1.13 (0.84–1.52)	0.425
Recessive			0.82 (0.61–1.10)	0.180	0.87 (0.65–1.18)	0.371
Additive			0.96 (0.81–1.15)	0.683^c^	0.96 (0.83–1.19)	0.955^c^
METTL14 rs1064034 A>T, HWE = 0.557						
TT	235 (51.3%)	239 (51.7%)	1.00		1.00	
AT	179 (39.1%)	183 (39.6%)	0.99 (0.76–1.31)	0.970	0.99 (0.75–1.31)	0.944
AA	44 (9.6%)	40 (8.7%)	1.12 (0.70–1.78)	0.636	1.18 (0.74–1.90)	0.491
Dominant			0.89 (0.57–1.40)	0.617	0.84 (0.53–1.33)	0.465
Recessive			0.98 (0.76–1.27)	0.898	0.98 (0.75–1.27)	0.864
Additive			0.97 (0.79–1.18)	0.750^c^	0.96 (0.78–1.17)	0.651^c^
METTL14 rs298982 G>A, HWE = 0.268						
GG	340 (74.2%)	377 (81.6%)	1.00		1.00	
GA	110 (24.0%)	82 (18.0%)	**1.47 (1.07**–**2.03)**	**0.019**	**1.46 (1.06**–**2.02)**	**0.022**
AA	8 (1.8%)	2 (0.4%)	4.44 (0.94–21.03)	0.084^d^	**6.11 (1.11**–**33.54)**	**0.037**
Dominant			**1.54 (1.12**–**2.11)**	**0.007**	**1.54 (1.12**–**2.12)**	**0.008**
Recessive			4.09 (0.86–19.36)	0.109^d^	**5.66 (1.03**–**31.15)**	**0.046**
Additive			**1.55(1.16**–**2.09)**	**0.003** ^c^	**1.57 (1.17**–**2.13)**	**0.003** ^c^
The combined effect of risk genotypes^e^						
0	131 (28.6%)	156 (33.8%)	1.00			
1	85 (18.5%)	125 (27.1%)	0.81 (0.57–1.16)	0.251	0.81 (0.56–1.17)	0.259
2	239 (52.2%)	180 (38.9%)	**1.58 (1.17**–**2.14)**	**0.003**	**1.57 (1.16**–**2.13)**	**0.004**
3	3 (0.7%)	1 (0.2%)	3.57 (0.37–34.76)	0.506^d^	3.89 (0.39–38.14)	0.243
0–1	216 (47.2%)	281 (60.8%)	1.00			
2–3	242 (52.8%)	181 (39.2%)	**1.74 (1.34**–**2.26)**	**0.000**	**1.73 (1.33**–**2.26)**	**0.000**

The noteworthy results were highlighted in bold.

^a^ χ2 test for genotype distributions between cases and controls.

^b^ Adjusted for age, gravidity, and parity.

^c^ Genotypes analyzed by the Cochran-Armitage test.

^d^ Genotypes analyzed by the Fisher’s exact test.

^e^ Risk genotypes were rs9884978 GA, rs6238061 GG, rs4834698 CT, rs1064034 AA, and rs298982 GA/AA.

Subsequently, the rs9884978 GA, rs6238061 GG, rs4834698 CT, rs1064034 AA, and rs298982 GA/AA genotypes were defined as risk genotypes based on their ORs, and their combined effect on the risk of ovarian endometriosis was tested. Participants with two risk genotypes were found to have a 57% increased risk of ovarian endometriosis (AOR = 1.57, 95% CI = 1.16–2.13, *P* = 0.004) and those with 2–3 risk genotypes were found to have a 73% increased risk of developing ovarian endometriosis when compared with those with 0–1 risk genotype (AOR = 1.73, 95% CI = 1.33–2.26, *P* = 0.000).

### 3.3 Stratification analysis

The associations between the risk genotypes of the selected SNPs and susceptibility to ovarian endometriosis were further evaluated in subgroups divided by age, gravidity, parity, and rASRM stage. As shown in Table 3, the rs298982 GA/AA genotype was significantly associated with increased ovarian endometriosis risk in the gravidity≤1 (AOR = 1.51, 95% CI = 1.07–2.12, *P* = 0.019), parity≤1 (AOR = 1.50, 95% CI = 1.07–2.10, *P* = 0.020), rASRM stage I (AOR = 1.46, 95% CI = 1.04–2.07, *P* = 0.031), and rASRM stage II + III + IV (AOR = 1.83, 95% CI = 1.15–2.91, *P* = 0.011) subgroups when compared with the GG genotype. Similarly, participants with 2–3 risk genotypes had a significantly increased risk of ovarian endometriosis in all subgroups, except for the gravidity>1 and parity>1 subgroups, when compared with those with 0–1 risk genotype. However, no significant association was found between the remaining risk genotypes and ovarian endometriosis risk in any subgroups.

**TABLE 3:** Stratification analysis of risk genotypes with ovarian endometriosis susceptibility.

Variables	rs9884978 G>A (case/control)		AOR (95% CI)	*P* ^a^	rs6238061 A>G (case/control)		AOR (95% CI)	*P* ^a^	rs298982 G>A (case/control)		AOR (95% CI)	*P* ^a^	Combined effect of risk genotypes (case/control)		AOR (95% CI)	*P* ^a^
	GA	GG/AA			GG	AA/AG			GA/AA	GG			0–1	2–3		
Age																
≤30	67/79	139/174	1.02 (0.68–1.52)	0.928	11/7	195/246	2.08 (0.78–5.56)	0.145	54/49	152/204	1.50 (0.97–2.34)	0.071	102/156	104/97	**1.63 (1.12**–**2.37)**	**0.011**
>30	90/66	162/143	1.29 (0.87–1.92)	0.212	10/7	242/202	1.16 (0.43–3.16)	0.767	64/36	188/173	1.59 (0.99–2.53)	0.051	114/125	138/84	**1.86 (1.27**–**2.72)**	**0.001**
Gravidity																
≤1	142/116	268/277	1.23 (0.91–1.65)	0.186	18/12	392/381	1.48 (0.69–3.17)	0.309	104/73	306/320	**1.51 (1.07**–**2.12)**	**0.019**	196/245	214/148	**1.76 (1.33**–**2.34)**	**0.000**
>1	15/29	33/40	0.86 (0.31–2.35)	0.762	3/2	45/67	0.63 (0.05–7.93)	0.718	14/12	34/57	2.29 (0.70–7.54)	0.170	20/36	28/33	1.59 (0.59–4.26)	0.352
Parity																
≤1	144/121	274/285	1.21 (0.90–1.63)	0.211	18/12	400/394	1.47 (0.69–3.13)	0.320	105/75	313/331	**1.50 (1.07**–**2.10)**	**0.020**	201/253	217/153	**1.74 (1.32**–**2.31)**	**0.000**
>1	13/24	27/32	1.13 (0.37–3.43)	0.828	3/2	37/54	0.68 (0.06–8.42)	0.762	13/10	27/46	1.98 (0.59–6.58)	0.267	15/28	25/28	1.58 (0.54–4.65)	0.408
rASRM																
I	118/145	221/317	1.18 (0.87–1.59)	0.289	14/14	325/448	1.39 (0.65–2.97)	0.399	83/85	256/377	**1.46 (1.04**–**2.07)**	**0.031**	160/281	179/181	**1.74 (1.31**–**2.33)**	**0.000**
II + III + IV	39/145	80/317	1.07 (0.69–1.66)	0.760	7/14	112/448	2.02 (0.79–5.18)	0.143	35/85	84/377	**1.83 (1.15**–**2.91)**	**0.011**	56/281	63/181	**1.73 (1.15**–**2.61)**	**0.009**

The noteworthy results were highlighted in bold.

^a^ Adjusted for age, gravidity, and parity.

### 3.4 Associations between METTL14 haplotypes and ovarian endometriosis risk

Next, we investigated whether the haplotypes of the five METTL14 gene SNPs are linked to ovarian endometriosis risk. As shown in Table 4, the haplotype AGTTG was linked with significantly decreased ovarian endometriosis risk (AOR = 0.55, 95% CI = 0.31–0.97, *P* = 0.039) compared with the reference haplotype GACAG. In contrast, the haplotype GATAA was significantly associated with increased ovarian endometriosis risk when compared with the reference haplotype (AOR = 5.54, 95% CI = 1.63–18.87, *P* = 0.006).

**TABLE 4:** Association between inferred haplotypes of the METTL14 gene and ovarian endometriosis risk.

rs9884978	rs6238061	rs4834698	rs1064034	rs298982	Cases (n = 916)	Controls (n = 924)	Crude OR (95% CI)	*P*	AOR (95% CI)	*P* ^a^
G	A	C	A	G	122	121	1.00		1.00	
A	A	T	T	A	29	17	1.69 (0.88–3.24)	0.112	1.75 (0.91–3.37)	0.093
A	A	T	T	G	132	141	0.93 (0.66–1.31)	0.674	0.97 (0.68–1.38)	0.873
A	G	T	T	G	23	44	**0.52 (0.30**–**0.91)**	**0.022**	**0.55 (0.31**–**0.97)**	**0.039**
G	A	C	T	G	335	336	0.99 (0.74–1.33)	0.940	1.00 (0.74–1.34)	0.980
G	A	T	A	A	16	4	**3.97 (1.29**–**12.21)**	**0.016** ^b^	**5.54 (1.63**–**18.87)**	**0.006**
G	A	T	A	G	72	86	0.83 (0.56–1.24)	0.364	0.88 (0.58–1.31)	0.518
G	A	T	T	A	57	42	1.35 (0.84–2.16)	0.217	1.32 (0.82–2.12)	0.256
G	G	T	A	A	22	23	0.95 (0.50–1.79)	0.871	1.15 (0.07–18.65)	0.921
G	G	T	A	G	31	28	1.10 (0.62–1.94)	0.747	1.15 (0.65–2.05)	0.628
G	G	T	T	G	69	80	0.86 (0.57–1.29)	0.454	0.86 (0.57–1.30)	0.485

The noteworthy results were highlighted in bold.

^a^ Adjusted for age, gravidity, and parity.

^b^ Analyzed by the Fisher’s exact test.

### 3.5 FPRP and BFDP values for all significant associations

To examine the statistical robustness of the findings and assess whether the statistically significant associations required further analysis, FPRP and BFDP analyses were conducted. The FPRP and BFDP values were calculated using 0.25, 0.1, and 0.01 as three levels of prior probabilities (Table 5). At a prior probability level of 0.25, all the significant associations were noteworthy (<0.8) in the BFDP test and 9 of the 11 significant associations were noteworthy (<0.2) in the FPRP test, with the exceptions being the associations of haplotype analysis. When assuming a prior probability of 0.1, only 3 of the 11 significant associations were noteworthy in both tests: the rs62328061 (AG vs. AA: crude OR = 0.68, 95% CI = 0.51–0.91, *P* = 0.010; FPRP value = 0.142, BFDP value = 0.626), the rs298982 (dominant model: crude OR = 1.54, 95% CI = 1.12–2.11, P = 0.007; FPRP value = 0.129, BFDP value = 0.607), and the combined effect of risk genotypes (2 vs. 0: crude OR = 1.58, 95% CI = 1.17–2.14, *P* = 0.003; FPRP value = 0.068, BFDP value = 0.478). However, when assuming a prior probability of 0.01, no noteworthy result was observed in any significant association using the FPRP or BFDP test.

**TABLE 5:** FPRP and BFDP analysises for significant findings.

	Crude OR^a^ (95% CI)	*P* ^b^	Statistical power^c^	Prior probability FPRP/BFDP^d^		
				0.25	0.1	0.01
**rs62328061**						
AG vs. AA	0.68 (0.51–0.91)	0.010	0.542	**0.052/0.358**	**0.142/0.626**	0.646**/**0.949
Dominant	0.74 (0.56–0.98)	0.035	0.822	**0.113/0.542**	0.277/**0.781**	0.808/0.975
**rs298982**						
GA vs. GG	1.47 (1.07–2.03)	0.019	0.555	**0.093/0.474**	0.235/**0.730**	0.772/0.968
Dominant	1.54 (1.12–2.11)	0.007	0.427	**0.047/0.340**	**0.129/0.607**	0.619/0.944
GA/AA vs. GG						
Gravidity ≤1	1.49 (1.06–2.09)	0.021	0.458	**0.111/0.493**	0.272/**0.745**	0.804/0.970
Parity ≤1	1.48 (1.06–2.07)	0.021	0.49	**0.109/0.498**	0.269/**0.749**	0.802/0.970
rASRM stage I	1.44 (1.02–2.02)	0.037	0.727	**0.113/0.557**	0.277/**0.791**	0.808/0.976
rASRM stage II + III + IV	1.85 (1.17–2.93)	0.009	0.18	**0.155/0.471**	0.355/**0.727**	0.858/0.967
Combined effect of risk genotypes						
2 vs. 0	1.58 (1.17–2.14)	0.003	0.373	**0.024/0.234**	**0.068/0.478**	0.444/0.910
Haplotype analysis						
AGTTG vs. GACAG	0.52 (0.30–0.91)	0.022	0.203	0.245/**0.600**	0.493/0.818	0.915/0.980
GATAA vs GACAG	3.97 (1.29–12.21)	0.016	0.036	0.57/**0.696**	0.799/0.873	0.978/0.987

The noteworthy results were highlighted in bold.

^a^ Crude OR, reported in Tables 2–4.

^b^ χ2test was used to calculate the genotype and haplotype frequency distributions.

^c^ Statistical power was calculated using the number of observations, crude OR, and *P* values.

^d^ FPRP<0.2 and BFDP<0.8 were considered noteworthy.

### 3.6 Effect of SNPs on gene expression

**FIGURE 1:**
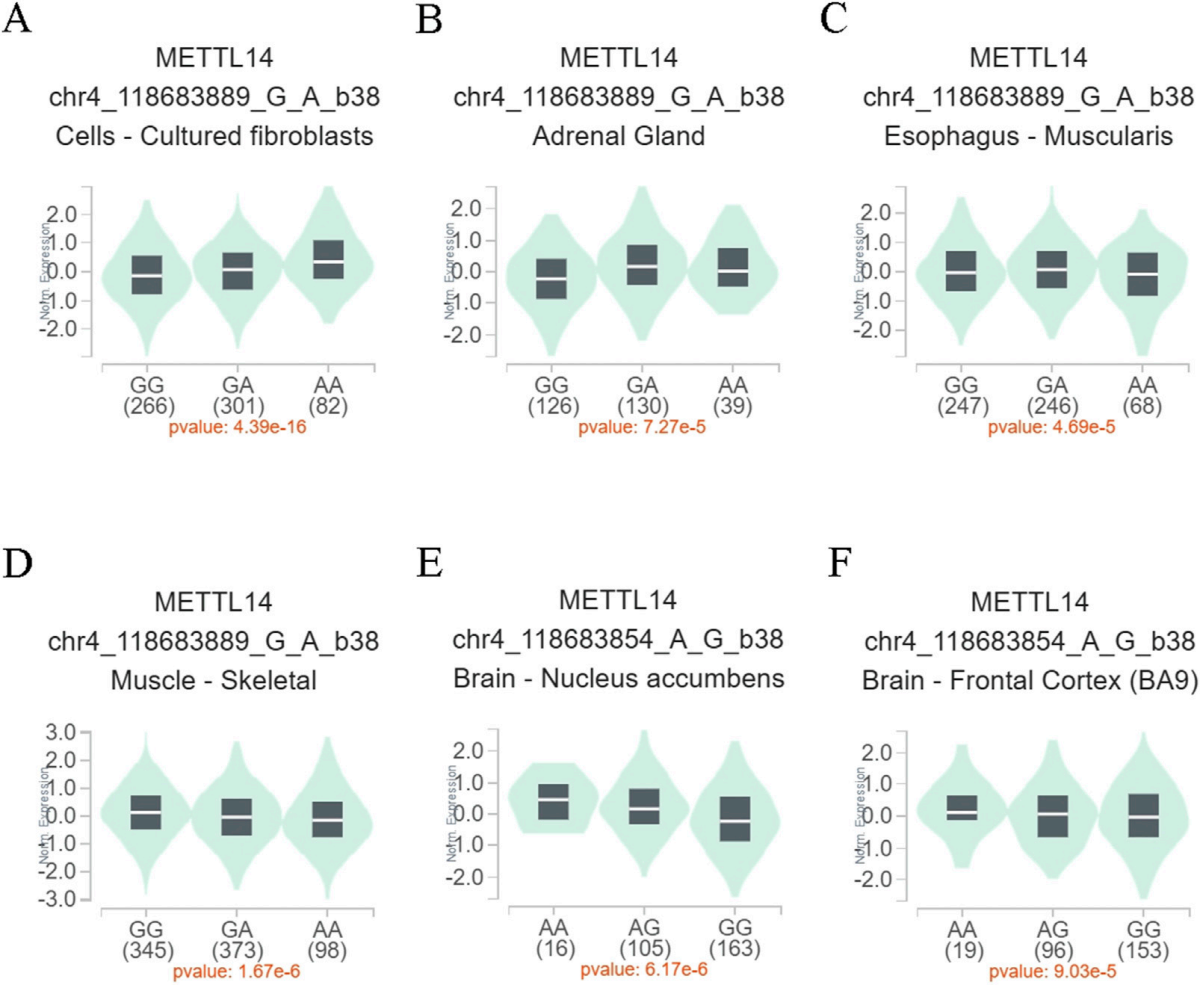
Functional relevance of rs9884978 and rs298982 on gene expression in GTEx database. **(A–D)** The genotype of rs9884978 and expression of METTL14 gene in different tissues. **(E, F)** The genotype of rs298982 and expression of METTL14 gene in the nucleus accumbens and frontal cortex.

To further assess whether the functional relevance of the METTL14 variant genotypes affects gene expression levels, eQTL analysis was performed to explore the effects of rs9884978 G>A and rs298982 G>A on gene expression. As shown in Figure 1, individuals with the rs9884978 GA/AA genotypes exhibited significantly higher levels of METTL14 mRNA compared to those with the rs9884978 GG genotype in cultured fibroblasts (*P* = 4.39 × 10^−16^) and adrenal glands (*P* = 7.27 × 10^−5^) (Figures 1A, B). In contrast, lower expression levels were observed in esophageal muscle (*P* = 4.69 × 10^−5^) and skeletal muscle (*P* = 1.67 × 10^−6^) (Figures 1C, D). Moreover, individuals with the rs298982 GA/GG genotypes showed significantly reduced levels of METTL14 mRNA compared to those with the rs298982 AA genotype in the nucleus accumbens (*P* = 6.17 × 10^−6^) and frontal cortex (*p* = 9.03 × 10^−5^) of the brain (Figures 1E, F).

## 4 Discussion

This study was motivated by the importance of m6A modification genes in endometriosis development. We hypothesized that polymorphisms in the METTL14 gene might affect susceptibility to endometriosis. Our investigation found that common variations in the METTL14 gene were notably linked to endometriosis risk among Chinese women. This research could improve our understanding of the biological and genetic factors underlying endometriosis.

The main RNA m^6^A methyltransferase complex (MTC) is composed of METTL3, METTL14, and WT1-associated protein (WTAP). Of these, METTL14 serves as an RNA-binding scaffold in MTC but does not catalyze methyl-group transfer (Liu et al., 2019). Recent studies have revealed that METTL14 acts as an oncogene or a suppressor in diverse human cancers (Guan et al., 2022; Liu et al., 2022). In accordance, Cui *et al.* found that knockdown of METTL14 substantially enhanced the incidence of tumorigenicity in glioblastoma stem cells (Cui et al., 2017). In contrast, Wang *et al.* reported that METTL14 overexpression promotes tumor cell migration and colony formation in pancreatic cancer and that METTL14 expression is significantly positively associated with poor survival (Wang et al., 2020a).

Regarding its role in endometriosis, studies have found that patients with endometriosis have lower levels of m^6^A in the endometrium than individuals with normal endometrium (Li et al., 2021) and that the expression levels of METTL3 and METTL14 are downregulated in ectopic endometria compared with eutopic endometria (Shen et al., 2023). In addition, knockdown of METTL3 or METTL14 has been found to facilitate the proliferation, migration, and invasion of human endometrial stromal cells (Li et al., 2021; Shen et al., 2023). These findings suggest that the upregulation of METTL3 or METTL14 expression in endometriosis has a favorable effect on patients with this disease.

Functional SNPs located in or near a gene may influence the gene’s function through impacting mRNA splicing, nucleo-cytoplasmic export, stability, translation, and many other factors (Albert, 2011; Mize and Evans, 2024). Recent studies have reported novel associations between the functional SNPs of the METTL14 gene and susceptibility to many human diseases, such as Wilms tumor (Zhuo et al., 2021), neuroblastoma (Zhuo et al., 2020), hepatoblastoma (Chen et al., 2022), acute lymphoblastic leukemia (Luo et al., 2021), and coronary heart disease (Sun et al., 2022). However, to our knowledge, no other study investigated the association between METTL14 genetic variants and endometriosis risk. Our case-control study was the first to explore the associations between the functional SNPs of the METTL14 gene and susceptibility to endometriosis. We found that the rs298982 A variant genotypes were significantly associated with increased ovarian endometriosis risk, particularly in the gravidity≤1, parity≤1, and rASRM stage subgroups. In contrast, we found that the rs9884978 A and rs62328061 G variant genotypes were associated with decreased ovarian endometriosis risk. It is worth noting that carriers with the haplotype AGTTG, which contains rs9884978A and rs62328061 G alleles, were found to have a 45% lower risk of ovarian endometriosis (AOR = 0.55, 95% CI = 0.31–0.97, *P* = 0.039). Moreover, the eQTL analysis indicated that the rs9884978 A allele increases, while the rs298982 G allele decreases the expression of METTL14 in some tissues (Figure 1). These findings suggest that higher levels of METTL14 may exert a protective effect in individuals with ovarian endometriosis.

Consistent with previous studies, our results support that the METTL14 level is reduced in the ectopic endometrium and is associated with an increased risk of endometriosis (Shen et al., 2023). In the light of the crucial role that METTL14 plays in MTC, METTL14 downregulation may decrease m^6^A levels. In fact, studies have reported observation of significantly downregulated m^6^A levels in eutopic endometria and ectopic endometriosis when compared with normal endometria (Li et al., 2021; Wang et al., 2023). Here, we found that the rs9884978 GA/AA genotypes were significantly associated with the increase of METTL14 expression in cultured fibroblasts and adrenal glands (Figures 1A, B), suggesting that the protection role of rs9884978 GA/AA genotypes may be positively linked to the increase of METTL14 mRNA level in endometriosis. Conversely, the risk associated with rs298982 GA/GG genotypes was positively correlated with a reduction in METTL14 mRNA levels in endometriosis. Indeed, we observed a significant negative correlation between rs298982 GA/GG genotypes and METTL14 expression in both the nucleus accumbens and frontal cortex (Figures 1D, F). These findings are consistent with research reports that high expression of METTL14 in endometriosis promotes cell proliferation, migration, and invasion (Lv and Li, 2024).

Interestingly, a recent study by Wan *et al.* reported that conditional deletion of Mettl3 in the reproductive tract of female mice resulted in infertility due to compromised uterine endometrium receptivity and decidualization (Wan et al., 2023a), underscoring the importance of m^6^A modifiers in the development of endometriosis. Notably, stratified analysis demonstrated that carriers of rs298982 GA/AA genotypes had a 50% increased risk of ovarian endometriosis in the parity≤1 subgroup, suggesting that the biological function of METTL14 gene might impact the risk of infertility. The potential biological functions and mechanisms of the METTL14 gene in the development of endometriosis and infertility deserve further exploration.

The findings of this study support the hypothesis that genetic polymorphisms in the genomic region of METTL14 may influence ovarian endometriosis risk. However, this study has several limitations that must be acknowledged. First, the participants were recruited from one center, which might have introduced selection and information bias due to non-representative subject selection and retrospective exposure data collection. Second, only five functional SNPs in the METTL14 gene were examined regarding their impact on endometriosis susceptibility despite the fact that a greater number of potential functional polymorphisms deserve investigation. Third, the impact of possible confounders on endometriosis risk, such as nutritional status, environmental factors, and sleep quality, could not be examined due to lack of patient data. Despite these limitations, our findings are the first to highlight the critical roles of METTL14 genetic variation in determining susceptibility to endometriosis among Chinese women. The observed associations are worth further validation in well-designed studies investigating large samples with participants from diverse populations. There is growing evidence that SNPs are closely linked to complex diseases. As a result, researchers require straightforward and reliable techniques that enable rapid, sensitive, and selective genotyping and quantification of SNPs for diagnosis. This study also indicates that SNPs could serve as a new generation of biomarkers for the early diagnosis and prognosis of endometriosis.

## Data Availability

The original contributions presented in the study are publicly available. This data can be found here: Hua, Wf. (2024). METTL14-SNPGenotypingData. 10.13140/RG.2.2.21891.62241. https://www.researchgate.net/publication/387021842_METTL14-SNPGenotypingData.
